# Correction for Grünebast et al., “Degradation of ribosomal RNA during *Plasmodium falciparum* gametocytogenesis”

**DOI:** 10.1128/mbio.01098-26

**Published:** 2026-09-04

**Authors:** Janne Grünebast, Ritwik Singhal, Robin Bromley, Sachie Kanatani, Kaylee Watson, Sophie Olson, Franck Dumetz, Tales Vicari Pascini, Abhai Tripathi, Julie C. Dunning Hotopp, Photini Sinnis, Manuel Llinás, David Serre

## AUTHOR CORRECTION

Volume 16, no. 11, e02565-25, 2025, https://doi.org/10.1128/mbio.02565-25. We recently discovered, after extensive genomic analyses, that one of the *P. falciparum* strains used in the study had been mislabeled in the laboratory: the strain cultured at UMSOM and referred to as NF54 is most likely a parasite derived from a Malawian field isolate (UMB-MWI) grown in parallel at our institution. The NF54 parasites cultured at PSU, JHSPH, and Sanaria have genotypes matching the reported NF54 alleles and are not affected by this authenticity problem. The uses of “NF54” requiring correction are listed below.

We have corrected the NCBI submission associated with these data. The results and the conclusions of the study remain valid. We apologize for the error.

Introduction, last paragraph, sentence 1: “NF54 parasites” should read “Malawi field isolate (MWI).”

Materials and Methods, “*P. falciparum* cultures and RNA isolation,” sentences 1 and 2: “NF54” should read “MWI.”

Materials and Methods, “NF54 culture and isolation protocols (UMSOM),” section heading and paragraph 1, sentence 1: “NF54” should read “MWI.”

Materials and Methods, “*In vitro* polyadenylation,” sentence 1: “NF54” should read “MWI.”

Materials and Methods, “Bioinformatic analyses,” last paragraph, sentences 2–4: “NF54” should read “MWI.”

Results, “Direct RNA sequencing enables characterization of the NF54 *P. falciparum* transcriptome,” sentence 1: “NF54” should read “MWI.”

Results, “rRNA degradation in *P. falciparum* NF54 gametocytes, section heading: “NF54” should read “MWI.”

Figure 1: “NF54” should read “MWI” for the Rings, Trophozoites, Schizonts, and Gametocyte V bars.

Figure 1 legend, sentence 1: “NF54” should read “NF54 and MWI”.

Figure 1 legend, sentences 2 and 3: “NF54” should read “MWI” and “salivary gland sporozoites” should read “NF54 salivary gland sporozoites.”

Figure 2b and d: “NF54” should read “MWI.”

Figure 2 legend, sentence 1: “NF54” should read “MWI.”

Table 1: “NF54” should read “MWI” throughout.

Figure 3: “NF54” should read “MWI” throughout.

Figure 3 legend, sentence 1: “NF54” should read “MWI.”

Results, “Replication and determination of the timing of rRNA degradation during gametocytogenesis,” paragraph 2, sentence 1: “the NF54 strain” should read “the MWI and NF54 strains.”

Results, “Replication and determination of the timing of rRNA degradation during gametocytogenesis,” paragraph 2, sentence 4, “NF54” should read “MWI.”

Figure 4: “NF54” should read “MWI” throughout.

Figure 4 legend, sentences 1 and 2: “NF54” should read “MWI.”

Results, “Replication and determination of the timing of rRNA degradation during gametocytogenesis,” paragraph 3, sentences 1 and 3, “NF54” should read “MWI.”

Figure 5a: “NF54” should read “MWI.”

Figure 5 legend, sentence 2: “NF54” should read “MWI.”

Supplemental material, Supplemental Figures: “NF54” should read “MWI” in Fig. S2B and D, 3A, and S4 and their legends. For Fig. S5, “NF54” should be removed from all panels and “stage V gametocytes replicate #2 (dark purple) compared to NF54 trophozoites” in the legend should read “NF54 stage V gametocytes replicate #2 (dark purple) compared to MWI trophozoites.” Corrected figures are given in the revised supplemental file in this correction.

Supplemental material, Table S2: “NF54” should read “MWI” in rows 4–7, 10, and 11. A corrected table is given in the revised supplemental file in this correction.

Supplemental material, Table S3: “NF54” should read “MWI” in rows 5–8. A corrected table is given in the revised supplemental file in this correction.

Supplemental material, Table S4: “NF54” should read “MWI” in rows 4–7, 10, and 11. A corrected table is given in the revised supplemental file in this correction.

## SUPPLEMENTAL MATERIAL

Supplemental Figures mbio.01098-26-s0001.pdf Complete supplemental figure file incorporating corrections.

Table S2 mbio.01098-26-s0002.xlsx Corrected Table S2.

Table S3 mbio.01098-26-s0003.xlsx Corrected Table S3.

Table S4 mbio.01098-26-s0004.xlsx Corrected Table S4.

ASM does not own the copyrights to Supplemental Material that may be linked to, or accessed through, an article. The authors have granted ASM a non-exclusive, world-wide license to publish the Supplemental Material files. Please contact the corresponding author directly for reuse.

